# Facial Hair Decreases Fit Factor of Masks and Respirators in Healthcare Providers

**DOI:** 10.3390/biology10101031

**Published:** 2021-10-12

**Authors:** Borja De-Yñigo-Mojado, Ricardo Becerro-de-Bengoa-Vallejo, Marta Elena Losa-Iglesias, Javier Madera-García, David Rodríguez-Sanz, Cesar Calvo-Lobo, Daniel López-López, María Teresa Angulo-Carrere, Marta San-Antolín

**Affiliations:** 1 Facultad de Enfermería, Fisioterapia y Podología, Universidad Complutense de Madrid, 28040 Madrid, Spain; borjadeynigo@gmail.com (B.D.-Y.-M.); ribebeva@ucm.es (R.B.-d.-B.-V.); davidrodriguezsanz@ucm.es (D.R.-S.); anguloca@ucm.es (M.T.A.-C.); 2 Faculty of Health Sciences, Universidad Rey Juan Carlos, 28933 Móstoles, Spain; marta.losa@urjc.es; 3 Department of direction, Staub Engineering, 33007 Oviedo, Spain; javiermadera@staubinge.es; 4 Research, Health and Podiatry Group, Department of Health Sciences, Faculty of Nursing and Podiatry, Universidade da Coruña, 15403 Ferrol, Spain; daniellopez@udc.es; 5 Department of Psychology, Universidad Europea de Madrid, 28670 Villaviciosa de Odón, Spain; marta.sanantolin@universidadeuropea.es

**Keywords:** community health workers, filtration, hair, masks, respiratory protective devices

## Abstract

**Simple Summary:**

This study supports evidence regarding the effects of facial skin hair on the fit factor of surgical masks and filtering respirators in healthcare providers. The fit factor of surgical masks does not seem to be modified by the presence of facial hair, showing a low fit factor in both bearded and non-bearded healthcare providers. In contrast, the use of filtering respirators in conjunction with the presence of facial hair impairs their fit factor. Regarding healthcare environments, sanitary personnel should use filtering respirators under non-bearded conditions.

**Abstract:**

In response to the current state of the COVID-19 pandemic, healthcare providers are using common surgical masks and filtering respirators in conjunction with the presence of facial hair, which could lead to a large number of particles passing into their respiratory system. The purpose of this study was to determine the fit factor effectiveness of filtering respirators and surgical masks in bearded versus non-bearded healthcare providers. A controlled randomized clinical trial (NCT04391010) was carried out, analyzing a sample of 63 healthcare providers. The fit factors of surgical masks and FFP3 filtering respirators for healthcare providers with (*n* = 32) and without (*n* = 31) facial hair were compared. Fit factors were measured during an exercises protocol in which healthcare providers wore surgical masks and FFP3 filtering respirators. Surgical mask fit factor comparisons did not show significant differences (*p* > 0.05) between healthcare providers with and without facial hair. In contrast, filtering respirator fit factor comparisons showed statistically significant differences (*p* < 0.01) between both groups, indicating that healthcare providers with facial hair showed lower fit factor scores, which implies a worse fit factor with respect to healthcare providers without facial hair. The fit factor effectiveness of filtering respirators was reduced in healthcare providers with facial hair. The authors of this paper encourage healthcare providers to trim their beards during filtering respirator use or wear full-mask filtering facepiece respirators, especially during the COVID-19 pandemic.

## 1. Introduction

Worldwide, in response to the COVID-19 pandemic, the use of surgical masks and filtering respirators by healthcare providers has become commonplace [1]. Healthcare providers are at the forefront of COVID-19 management and face a highly probability of being infected. As the most common transmission route seems to be via droplet and aerosol inhalation, the use of masks and filtering respirators may be considered a first-line prevention strategy for healthcare providers [2].

While surgical masks do not seem to be sufficiently effective in virus dissemination, as it is unlikely that they prevent the entry of contaminated aerosols into the respiratory system [3], filtering respirators provide better protection against aerosol and droplet penetration, as a seal around the nose and mouth is created [2]. Filtering face piece (FFP) N95 respirators seem to be the most commonly used respirators, providing a tight fit with a particle filtering effectiveness of more than 95% regarding a median of particles size of 0.3 μm [2,4].

Currently, Centers for Disease Control and Prevention encourage healthcare providers who are commonly exposed to organic aerosols to use filtering respirators. Thus, the fit factors of surgical masks and filtering respirators in healthcare providers should be accurately determined. In addition, the filtering capacity of surgical masks seems to be poor, even when using several surgical masks simultaneously [5,6,7,8]. According to the medical community as a whole, Centers for Disease Control and Prevention have determined unsafe practices in healthcare providers at risk of suffering from viral infections. Therefore, infection prevention should be considered a priority strategy in clinical settings [9].

According to the above, the fit factor was defined as an individual quantitative filtration rate for a particular respiration device. This measurement may estimate the concentration ratio of particles located in ambient air in comparison to the concentration rate of particles inside the respirator device when worn. The main function that determines a mask’s filtering capacity has been determined as the ability of this device to form a tight seal with a healthcare provider’s face, avoiding air leakage between both the healthcare provider’s face and the mask’s edges [10]. Indeed, several fit tests types have been proposed to assess the capacity of face seal masks, resulting in a numerical value called the fit factor, which may accurately determine a mask’s facial fit ability [10,11,12,13].

Over three decades ago, it was claimed that the presence of a beard increases leakage through filtering respirator face seals, and it was thus suggested that wearing respirators in conjunction with the presence of facial hair could be a risk factor for leakage [14]. Nevertheless, facial hair seems to be commonly present in male workers, with up to 49% of male healthcare providers maintaining a beard, and there appears to be a correlation between a reduced respirator fit factor and an increase in facial hair [15]. Indeed, beard length as well as areal density may negatively influence the fit factor, although respirator fit tests may achieve an adequate fit factor even with the presence of substantial facial hair in the seal area [16]. On the contrary, bearded healthcare providers who wear surgical masks do not seem to present an increase in bacterial shedding likelihood with respect to non-bearded healthcare providers [17]. These controversial findings regarding the fit factor of filtering respirators and the use of masks by healthcare providers with and without facial hair need further investigation. Moreover, the different effects that the presence or absence of facial hair can have currently remain unclear, especially in the context of the COVID-19 pandemic [2].

According to these findings, we hypothesized that the presence of facial hair in healthcare providers would decrease the fit factor of filtering respirator use more so than that of surgical mask use. Thus, the purpose of this study was to determine the fit factor effectiveness of filtering respirators and surgical masks in bearded versus non-bearded healthcare providers.

## 2. Materials and Methods

### 2.1. Study Design

A controlled, randomized clinical trial study was carried out among healthcare providers from the Asturias Principality, Spain, from May 2020 to June 2020, according to the Consolidated Standards of Reporting Trials (CONSORT) criteria [18]. After completing the study, a sample of 63 healthcare providers with (*n* = 32) and without (*n* = 31) facial hair was analyzed. The fit factor was measured during an exercises protocol to determine particle count in surgical masks and FFP3 filtering respirators compared to particle count outside of surgical masks and filtering respirators, and comparisons between healthcare providers with and without facial hair were also made.

### 2.2. Ethical Considerations and Trial Registry

This controlled. Randomized clinical trial was performed according to the Declaration of Helsinki [19]. The Rey Juan Carlos University Ethics Committee (Madrid, Spain) approved this study with the 09/2015 registry code. This study was first prospectively registered at ClinicalTrials.gov with the clinical trial identifier NCT04391010. Before beginning the study, all healthcare providers were fully informed about the study procedures and provided signed informed consent.

### 2.3. Sample Size Calculation

G*Power^©^ version 3.1.9.2 statistical software (from Dusseldorf University, Dusseldorf, Germany) was applied to calculate the sample size using the Wilcoxon–Mann–Whitney test for 2 sample means according to non-parametric data distribution [20]. In order to achieve a large size effect for between-groups differences [21], an effect size with a Cohen *d* of 0.80 was used. In addition, an error probability for α of 0.05, a confidence interval (CI) of 80%, a power for 1-β error probability of 0.80, a two-tailed hypothesis, and an allocation ratio for N2/N1 of 1 were used. As a result, a total sample size of 54 healthcare providers, 27 healthcare providers with facial hair and 27 healthcare providers without facial hair, was obtained. According to a possible 15% loss to follow-up, a total sample size of 62 healthcare providers was required.

### 2.4. Participants, Recruiting, and Randomization

**Figure 1:**
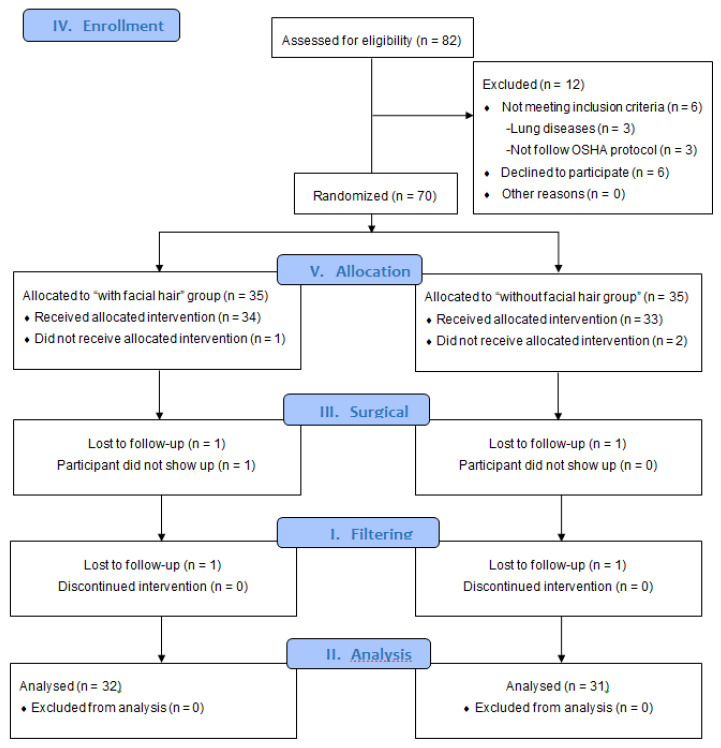
CONSORT [18] flow diagram. Abbreviations: OSHA, Occupational Safety & Health Administration.

From a total of 82 men recruited from the Asturias Principality and assessed for eligibility (Figure 1), a sample of 63 healthcare providers were randomly assigned and analyzed in an experimental group composed of healthcare providers with facial hair (*n* = 31) and a control group without facial hair (*n* = 32). Inclusion criteria were male healthcare providers older than 18 years old. Exclusion criteria were healthcare providers with lung diseases, lack of provision of informed signed consent, and healthcare providers who did not carry out healthcare according to Occupational Safety & Health Administration (OSHA) recommendations [22].

### 2.5. Descriptive Data

Descriptive data included weight (kg) assessed by a digital device (Bosch; AxxenceSlim-Line-model; Gerlingen, Germany); height (cm) evaluated by a tape (M807-20-model, Brueder-Mannesmann-Werkzeuge; Remscheid, Germany); and length of the face (mm), depth of the face (mm), width (mm) of the face, and width of the mouth (mm) assessed by a compass device (Staedtler; Mars-basic-554-model; Nüremberg, Germany) [10,22].

### 2.6. Surgical Mask and Filtering Respirators

The fit factor of the surgical masks used by healthcare providers was measured and then compared with that of filtering respirators. FPP3 filtering respirators are considered as the most efficacious FFP filtering respirators in order to avoid bacteria and virus exposure. The proposed FPP3 filtering respirator types were Moldex-2505 (Culver-City, CA, USA), Aura-9332+ (3M-St Paul; Maplewood, MN, USA), and K-113 (3M-St Paul; Maplewood, MN, USA) [10,11,12,13,23]. Surgical masks (Shell type) were considered as the control masks commonly used by healthcare providers [17].

### 2.7. Fit Factor Procedure

The fit factors of surgical masks and FPP3 filtering respirators for healthcare providers with and without facial hair were compared [14,15,17]. According to the recommendation of the Occupational Safety & Health Administration (OSHA) [22], the determination of fit factors is deemed the gold standard procedure in order to quantify the filtration capacity of these filtering respirators and surgical masks, and the fit factor is an individual quantitative rate that indicates the filtration capacity of a specific respiration tool, such as surgical masks and filtering respirators. Indeed, this factor determines the particle concentration located in ambient air with respect to the particle concentration inside surgical masks or filtering respirators when worn, thereby allowing for the ability of these devices to maintain a tight seal with the healthcare provider’s face and prevent air leakage between the mask’s edges and the healthcare provider’s face to be examined [10,24].

According to the protocol described in previous studies [11,12,25,26,27] and OSHA recommendations [22], the fit factor has been considered as a quantitative tool that can be used to determine particle counts in surgical masks and filtering respirators with respect to particle counts outside of these respiratory devices. In the current study, healthcare providers performed an exercise protocol that comprised 8 exercises (Table 1). The fit testing procedure measured the difference between the ambient concentration particle count and the filtered concentration particle count inside the mask or filtering respirator. All types of particles with a size range from 0.02 to >1 μm [28] were assessed in a 15 m^2^ clean room, showing an approximate ambient concentration count from 2000 to 2500 particles [25,29]. Firstly, the fit factor was determined in healthcare providers with and without facial hair when using surgical masks. Secondly, the fit factor was determined in healthcare providers with and without facial hair when using FPP3 filtering respirators.

According to fit factor analysis, an adjusted quantitative analysis was carried out using a reliable tool (PortaCount^®^ Pro + Model-8038) [28], which was previously calibrated. This tool determined the count of particles with a size varying from 0.02 to 1 μm. From this device, two catheters were provided, and the longest catheter was connected to the surgical mask or filtering respirator using a leak-proof kit, which included adapters and catheters (TSI; Tsi-Inc; St-Paul, MN, USA). The catheter transducer was applied between the mouth and nose, placed at 5 mm with respect to the mask interior surface and 10–15 mm from the healthcare provider’ mouth, maintaining an air sample inside the surgical mask or filtering respirator. Furthermore, all assessments were performed in a clean room with an approximate surface size of 15 m^2^ [25,29].

With reference to previous research studies [11,12,25,26,27] and OSHA recommendations [22], the total fit factor was determined by a global fit factor adjusted by an estimated mean of 8 exercises, detailing the particle rate that a healthcare provider may inhale through a formula that included “N” as the performed number of exercises and “FFn” as the fit factor calculated in a specific exercise number:

Fit factor total (FFT) = N/[(1/FF1) + (1/FF2) + (1/FF3) + … + (1/FFn-1) + (1/FFn)]

### 2.8. Statistical Analysis

According to quantitative data, normality analyses were carried out using the Shapiro–Wilk test. The quantitative data are expressed as mean ± standard deviation (SD), median and interquartile ranges, and lower and upper limits for a 95% CI. Comparisons between groups were performed using the Mann–Whitney U test for non-parametric data and the independent Student t test for parametric data. Statistical analyses were performed using SPSS software 23.0 version (IBM SPSS Statistics; Windows; IBM Corp; Armonk, NY, USA). For all analyses, a *p*-value *<* 0.05 for a 95% confidence interval (CI) was considered as statistically significant.

## 3. Results

### 3.1. Flow Diagram and Descriptive Data

From the total of 82 healthcare providers assessed for eligibility (Figure 1), 6 healthcare providers did not meet the inclusion criteria and were excluded due to lung diseases (*n* = 3) and not following OSHA recommendations (*n* = 3), and another 6 healthcare providers declined to participate. Thus, the remaining 70 healthcare providers were randomly assigned to an experimental group of healthcare providers with facial hair (*n* = 35) and a control group without facial hair (*n* = 35). In addition, three healthcare providers did not receive allocated intervention, and four healthcare providers were lost to follow-up. Finally, a sample of 63 healthcare providers with (*n* = 32) and without (*n* = 31) facial hair was analyzed per the protocol. Table 2 showed descriptive data of the study sample. In addition, all data did not show normal distribution (*p* < 0.05), except for weight and height (*p* > 0.05).

### 3.2. Surgical Mask Fit Factor Comparisons

As displayed in Table 3, surgical mask fit factor comparisons did not show statistically significant differences (*p* > 0.05) between healthcare providers with and without facial hair in terms of total scores and scores for each exercise protocol.

### 3.3. Filtering Respirator Fit Factor Comparisons

As displayed in to Table 4, filtering respirator fit factor comparisons showed statistically significant differences (*p* < 0.01) for both groups, indicating that healthcare providers with facial hair provided lower total and protocol exercise fit factor scores than those of healthcare providers without facial hair, suggesting a worse fit factor with respect to healthcare providers without facial hair.

## 4. Discussion

Currently, surgical masks and filtering respirators have become key personal protective equipment devices for healthcare providers to avoid virus exposure via droplet and aerosol inhalation during the COVID-19 pandemic [1,2]. According to the World Health Organization recommendations for respiratory aerosol exposure during the COVID-19 pandemic, FFP3 filtering respirator use is the first-line prevention strategy [30]. Our study findings support the use of FPP3 filtering respirators to provide healthcare providers with a better fit factor than that of surgical masks. In addition, these findings are in accordance with previous research studies [11,12,25,26,27]. Moreover, to the best of our knowledge, this is the first study to be performed following the exercise protocol according to OSHA recommendations [22].

Healthcare providers who wore respirators in conjunction with facial hair showed a worse fit factor than that of healthcare providers without facial hair. Facial hair produced alterations to the face seal when using negative pressure respirators. The fit factor of half-mask respirators on bearded faces showed a median fit factor of 12 with 8% leakage, while the fit factor of half-mask respirators on clean-shaven faces showed a median fit factor of 2950 with 0.03% leakage. In addition, in a previous study, full-facepiece respirators on bearded faces provided a median fit factor of 30 with 3% leakage, while full-facepiece respirators on clean-shaven faces provided a median fit factor higher than 10,000 with less than 0.01% leakage [14]. In another study, facial hair was present in half of the participating male healthcare providers, and achieving an adequate respirator fit was hindered by the presence of facial hair [15]. Indeed, the fit factor of filtering respirators notably decreased when beard length was longer than 0.125 inches. Beard areal density and length, but not coarseness, have been deemed as predictors of the filtering respirator fit factor [16]. However, in another study, regarding the likelihood of bacterial shedding with use of surgical masks, differences were not observed between bearded healthcare providers, with 1.6 CFU, and clean-shaven healthcare providers, with 1.2 CFU [17]. The findings of these studies are in line with ours, suggesting that the fit factor effectiveness of filtering respirators is lower in bearded healthcare providers than in clean-shaven health providers, but the same is not true for surgical masks.

In the present study, Table 3 presents a high standard deviation and a low median value for the fit factor of surgical masks in the group without facial hair. This issue may be caused by the cranial shape and facial anatomy of the individuals in conjunction with the low fit factor observed in healthcare providers who wore surgical masks [11,12,25,27].

In a recent study, Hao et al. [31] examined the effectiveness of 136 filtering facepiece respirators of the N95-type and revealed that a significant percentage of the respirators fell notably below their claimed performance level. The fit factor differences of the different respirators used in this study were not analyzed due the sample size not being adequate to compare these three conditions, but these differences could have influenced our study findings. 

### 4.1. Clinical Recommendations

The authors encourage healthcare providers to maintain clean-shaven faces during the use of filtering respirators in order to achieve an adequate fit factor. Another option may be to wear full-mask filtering facepiece respirators. Despite surgical masks not showing fit factor differences between bearded and non-bearded healthcare providers, filtering respirators provide a better fit factor than that of surgical masks, and, as such, prevention strategies should be established and implemented on this basis, especially during the COVID-19 pandemic [2].

In a recent review, Howard et al. [32] discussed the use of masks by the public as source control for COVID-19 and pointed out the need for more randomized clinical trials. Despite the present study being carried out in healthcare environments, the authors highlight the potential impact of our findings on policy making for non-medical settings, as the results indicate that personnel of retail stores and other non-clinical environments should use filtering respirators under clean-shaven conditions.

### 4.2. Limitations and Future Research Lines

Some limitations may be considered in this research study. First, this study followed an observational research design. Future randomized clinical trials should include cross-over designs as well as masking procedures for outcome assessors and participants. Second, larger sample sizes could allow for the development of fit factor prediction models under bearded and non-bearded conditions. Lastly, future study designs should examine the test performance of the same subjects with a beard and after shaving in order to evaluate the differences in fit factors with the presence and absence of a beard. This issue may be considered as a weakness of the present study. In addition, beard length and areal density were not measured in the present study, and further studies should include these parameters as possible predictors of fit factors [16].

A report on the situation of COVID-19 in Spain dated 14 May 2020, established a significant difference (*p* < 0.001) between women and men admitted to intensive care units of 2.9% and 8%, respectively [33]. According to our research findings, we suggest that the highest percentage of affected patients may be partially due to men having facial hair and, which, consequently, may have lowered the filtering factor of facial masks and filtering respirators. Thus, further epidemiologic research is needed in order to clarify if this rate may be related to a worse fit factor of filtering respirators used by male healthcare providers with facial hair.

## 5. Conclusions

The fit factor effectiveness of filtering respirators was reduced in healthcare providers with facial hair. The authors of this paper encourage healthcare providers to trim their beards during filtering respirator use or wear full-mask filtering facepiece respirators, especially during the COVID-19 pandemic.

## Figures and Tables

**Table 1:** Exercise protocol to measure fit factor.

Number of Exercise	Name of Exercise	Description of Exercises (1 min Per All Exercises, Except for Number 6, Which Required 15 s)
1	Usual breathing	HCP was quiet with normal breathing
2	Deep breathing	HCP performed deep and large respirations to simulate breathing when great efforts are required
3	Neck lateral flexion	Usual breathing while neck was lateral flexed and muscles were stretched
4	Speaking out loud	HCP spoke out loud, counting from 0
5	Head flexo-extension	Usual breathing during head flexo-extension
6	Grimace	HCP frowned or smiled for 15 s
7	Trunk flexion	HCP carried out trunk flexion in order to touch their toes
8	Usual breathing	As in exercise 1, HCP was quiet with normal breathing

Abbreviations: HCP, healthcare providers.

**Table 2:** Descriptive data of the study sample with/without facial hair (*n* = 70).

Variables	Without Facial Hair (*n* = 35)		With Facial Hair (*n* = 35)		*p* Value
Descriptive Data	Mean ± SD (IC95%)	Median (IR)	Mean ± SD (IC95%)	Median (IR)	
Age (years)	34.43 ± 7.81 (31.61–37.25)	32.00 (6.00)	32.81 ± 5.52 (30.78–3.83)	33.00 (7.00)	0.539 **
Weight (kg)	70.46 ± 12.22 (66.06–74.87)	70.00 (15.25)	69.71 ± 11.53 (65.48–73.94)	69.00 (17.00)	0.762 *
Height (m)	1.70 ± 0.06 (1.67–1.73)	1.70 (0.12)	1.70 ± 0.09 (1.66–1.73)	1.74 (0.15)	0.994 *
BMI	24.15 ± 3.23 (22.99–25.32)	24.34 (3.46)	23.87 ± 2.56 (23.67–24.81)	23.62 (2.46)	0.531 **
Face length	111.62 ± 7.85 (108.79–114.45)	111.00 (9.00)	116.58 ± 8.44 (113.48–119.67)	115.00 (12.00)	0.021 **
Face depth (mm)	121.34 ± 6.64 (118.94–123.73)	121.20 (7.75)	126.481 ± 7.55 (123.71–129.25)	33.00 (7.00)	0.004 **
Face width	130.84 ± 6.62 (128.45–133.23)	130.00 (8.00)	139.58 ± 7.49 (136.83–142.33)	140.00 (8.00)	<0.001 **
Mouth width (mm)	47.81 ± 4.09 (46.33–49.28)	48.00 (5.75)	49.32 ± 4.37 (47.71–50.92)	49.00 (6.00)	0.260 **

Abbreviations: BMI, body mass index; CI, confidence interval; SD, standard deviation; IR, interquartile range; * *p* value from independent Student t test; ** *p* value from Mann–Whitney U test.

**Table 3:** Fit factor comparisons for exercises and total scores between groups with and without facial hair using surgical masks.

	Without Facial Hair with Surgical Masks (*n* = 32)		With Facial Hair with Surgical Masks (*n* = 31)		*p* Value *
Exercise	Mean ± SD (CI)%	Median (IR)	Mean ± SD (CI)%	Median (IR)	
Exercise 1	9.59 ± 27.03 (0.15–19.34)	2.22 (1.22)	2.46 ± 0.82 (2.16–2.77)	2.30 (1.10)	0.890
Exercise 2	8.04 ± 17.83 (1.60–14.47)	2.25 (1.75)	2.55 ± 0.85 (2.23–2.86)	2.30 (1.10)	0.659
Exercise 3	7.56 ± 17.80 (1.15–13.98)	2.10 (1.10)	2.38 ± 0.83 (2.07–2.68)	2.20 (0.80)	0.984
Exercise 4	6.20 ± 13.99 (1.16–13.99)	2.25 (0.80)	2.45 ± 0.75 (2.17–2.72)	2.20 (0.80)	0.962
Exercise 5	10.28 ± 26.28 (0.79–19.74)	3.00 (1.60)	3.22 ± 1.73 (2.58–3.86)	2.90 (1.20)	0.630
Exercise 6	3.15 ± 3.35 (1.95–4.36)	2.05 (1.10)	2.27 ± 0.93 (1.93–2.61)	1.90 (1.30)	0.385
Exercise 7	4.02 ± 6.53 (1.66–6.37)	1.90 (0.67)	2.09 ± 0.67 (1.85–2.34)	2.00 (0.70)	0.934
Exercise 8	5.55 ± 11.87 (1.27–9.83)	2.05 (0.92)	2.19 ± 0.70 (1.93–2.45)	2.10 (0.70)	0.563
Total score	4.68 ± 7.52 (1.97–7.39)	2.20 (0.97)	2.37 ± 0.73 (2.10–2.64)	2.20 (1.00)	0.788

Abbreviations: CI, confidence interval; SD, standard deviation. * Mann–Whitney U test for independent groups. *p* < 0.05 was considered as statistically significant for a 95% CI.

**Table 4:** Fit factor comparisons for exercises and total scores between groups with and without facial hair using FFP3 filtering respirators.

	Without Facial Hair with FFP3 Respirator (*n* = 32)		With Facial Hair with FFP3 Respirator (*n* = 31)		p * Value
Exercise	Mean ± SD (IC)%	Median (IR)	Mean ± SD (IC)%	Median (IR)	
Exercise 1	197.31 ± 203.31 (124.00 ± 270.61)	131.50 (172.50)	46.70 ± 59.55 (24.86 ± 68.55)	17.00 (57.30)	<0.01
Exercise 2	142.78 ± 111.05 (102.74 ± 182.81)	96.00 (149.75)	52.99 ± 75,58 (25.23 ± 80.75)	27.00 (73.20)	<0.01
Exercise 3	133.43 ± 92.34 (100.14 ± 166.73)	117.00 (121.71)	53.80 ± 61.98 (31.06 ± 76.54)	21.00 (81.00)	<0.01
Exercise 4	117.35 ± 90.53 (84.71 ± 150.00)	92.50 (117.75)	47.89 ± 54.02 (28.08 ± 67.61)	22.00 (72.00)	<0.01
Exercise 5	84.50 ± 49.57 (66.82 ± 102.37)	80.50 (87.25)	40.08 ± 27.62 (29.94 ± 50.21)	33.00 (23.00)	<0.01
Exercise 6	48.32 ± 40.97 (33.54 ± 63.09)	35.50 (58.75)	20.69 ± 19.97 (13.36 ± 28.01)	11.00 (28.00)	<0.01
Exercise 7	55.28 ± 35.99 (42.30 ± 68.25)	49.50 (51.00)	36.86 ± 43.89 (20.76 ± 52.96)	17.00 (54.60)	0.005
Exercise 8	115.31 ± 105.07 (77.42 ± 153.19)	88.00 (87.00)	51.69 ± 57.89 (30.45 ± 72.82)	24.00 (82.30)	<0.01
Total score	65.75 ± 37.58 (52.20 ± 79.29)	57.50 (51.75)	30.59 ± 29.98 (19.59 ± 41.58)	18.00 (38.30)	<0.01

Abbreviations: CI, confidence interval; SD, standard deviation. * Mann –Whitney U test for independent groups. *p* < 0.05 was considered as statistically significant for a 95% CI.

## Data Availability

The raw data are available upon request to the corresponding author.

## Abbreviations

CI	confidence interval
CONSORT	Consolidated Standards of Reporting Trials
FFP	filtering face piece
OSHA	Occupational Safety & Health Administration
SD	standard deviation
